# The detection of lung cancer using massive artificial neural network based on soft tissue technique

**DOI:** 10.1186/s12911-020-01220-z

**Published:** 2020-10-31

**Authors:** Kishore Rajagopalan, Suresh Babu

**Affiliations:** Department of Electronics and Communication Engineering (ECE), Kamaraj college of engineering and technology (Autonomous), Virudhunagar, India

**Keywords:** X-ray, Sensitivity, Lung cancer, Subtle, Accuracy

## Abstract

**Background:**

A proposed computer aided detection (CAD) scheme faces major issues during subtle nodule recognition. However, radiologists have not noticed subtle nodules in beginning stage of lung cancer while a proposed CAD scheme recognizes non subtle nodules using x-ray images.

**Method:**

Such an issue has been resolved by creating MANN (Massive Artificial Neural Network) based soft tissue technique from the lung segmented x-ray image. A soft tissue image recognizes nodule candidate for feature extortion and classification. X-ray images are downloaded using Japanese society of radiological technology (JSRT) image set. This image set includes 233 images (140 nodule x-ray images and 93 normal x-ray images). A mean size for a nodule is 17.8 mm and it is validated with computed tomography (CT) image. Thirty percent (42/140) abnormal represents subtle nodules and it is split into five stages (tremendously subtle, very subtle, subtle, observable, relatively observable) by radiologists.

**Result:**

A proposed CAD scheme without soft tissue technique attained 66.42% (93/140) sensitivity and 66.76% accuracy having 2.5 false positives per image. Utilizing soft tissue technique, many nodules superimposed by ribs as well as clavicles have identified (sensitivity is 72.85% (102/140) and accuracy is 72.96% at one false positive rate).

**Conclusion:**

In particular, a proposed CAD system determine sensitivity and accuracy in support of subtle nodules (sensitivity is 14/42 = 33.33% and accuracy is 33.66%) is statistically higher than CAD (sensitivity is 13/42 = 30.95% and accuracy is 30.97%) scheme without soft tissue technique. A proposed CAD scheme attained tremendously minimum false positive rate and it is a promising technique in support of cancerous recognition due to improved sensitivity and specificity.

## Background

### General

Cells (https://www.cancer.net/) were vital units in our lung region, which were having its unique framework. Cancer (https://www.healthline.com) is a syndrome which might appear as an increased abnormal cell uncontrollably. However, it happens across any portion of a body [1]. So, it results in the change in genetic behavior [1] which deter the regular flow (cell may fabricate new cells during early stages and it dies while they were growing old). It might have a possibility for producing cancer in the lymphatic system. Doctors partition cancer into categories based on its foundation. The categories were listed as Carcinomas, Sarcomas, Leukemias as well as Lymphomas (https://www.medicalnewstoday.com/articles/323648) [1].

The most significant utility for lung was holding a stream utilizing oxygen within the entire body. However, blood flow was interrupted through these cancer. Lung cancer is a single hazardous syndrome, which might present in small as well as non small cell [2]. It is a prime cause for both genders in many countries. Early detection has high endurance rate. But, it is usually noticed late due to the lack of symptom in its early phases [3].

Lung cancer recognition (moziani.tripod.com) in premature phase has no symptom. However, during this phase, the root cause has not well known. Once doctors discovered root cause has been ignored by the patient, which result in late diagnosis and further treatment. Lung cancer endurance prolongs to fall. Hence, 7.6% males as well as 11.3% females are net survival predicted during 2013–2017 [4] and it is indicated in Table 1.

**Table 1:** Lung cancer standardised one, five and 10 year net survival (2013–2017)

Sex	Years after diagnosis	Number of cases	Net surivival (%)
Female	1 year	85,270.0	44.5
Male	1 year	98,357.0	37.1
Persons	1 year	183,627.0	40.6
Female	5 years	85,270.0	19.0
Male	5 years	98,357.0	13.8
Persons	5 years	183,627.0	16.2
Female	10 years	134,006.0	11.3
Male	10 years	157,189.0	7.6
Persons	10 years	291,195.0	9.5

Nodules seen within an x-ray image might not essentially be lung cancer, it reports an abnormality which was specified as pneumonia, tuberculosis or calcified granuloma. So, it was a tedious work for radiologists during the past few decades. Lung nodule widens towards the chest center since natural lobe situated across the lung region needs to be known earlier [5]. However, it is a exigent task of radiologists since ribs and clavicles are being overlapped with it.

X-ray utilize few energy with direction in obtaining imagery rooted in body’s interior structure. They are frequently accustomed toward assisting with identifying cracked bone, glance for wound or infection and to find a strange object in soft tissue. These might utilize an iodine-based contrast material or barium to build up the visibility of specific organ, blood vessels, tissues or bone. So, it has been used to identify chest syndromes because they are most cost-effective, routinely available and dose-effective diagnostics. However, X-ray images are suited for the improvement done in the image processing technique which does not need iodine-based contrast material to pick up the visibility of a specific organ. Hence, 30% nodules in.

x ray image are missed by radiologists and that, 82–95% missed nodules are partly obscured by overlying bone such as ribs and clavicles [6, 7]. For solving the issue of detecting nodule which is overlapping with ribs and clavicles, we proposed a novel CAD scheme of MANN based soft tissue technique.

### Related works

J.S. Lin [8] used two level neural classifiers for reducing false positive through computer aided analysis. However, lung cancer was recognized [9]. A co-occurrence matrix using texture measures [10] has been employed in support of malignant nodule recognition.

Most ordinary problems encountered throughout nodule finding was overlapping rib and clavicle with a nodule. Existing computer aided detection scheme known as most efficient tool since it was missing lung nodule due to overlapping rib. When we utilize an overlapped image, it was difficult in detecting a suspicious area. Several imaging techniques have been proposed during recent literature review such as analyzing texture, watershed segmentation [11], Gaussian filters [12], active shape modeling [13] and quasi-Gabo filters [14].

In [15], feature sets hold translation invariant wavelet with co-occurrence mammogram attributes were used in image categorization. Features extracted from multi scale Gaussian filter bank and some specific features that were readily calculated from blob detector scheme to detect nodules [16]. Local curvature using image data was considered when viewed using relief map [17].

Matsumoto et al., proposed computer aided detection scheme using x-ray images at 11 false positive rates, even though the system had 80% sensitivity. But lung nodule detection accuracy was not improved [18]. Feng Li et al. [19, 20] detects small lung cancers in x-ray image for false positive reduction. This would increase their confidence level of radiologist by utilizing dual energy subtraction technique. However, using such technique requires specialized equipments and dual energy images are prone to motion artifact.

To address this problem, dual energy subtraction strategies using radiation exposures [21] were considered for decomposing a radiograph into bone-free and soft tissue free image. So, it had been widely accepted in clinical practice because its clinical value can improve diagnostic efficiency. However, there were problems such as high radiation dose and motion artifacts due to double exposure with different energies. This problem had been addressed by using deep learning [22]. Deep Learning has assumed that there was a nonlinear relationship between dual energy image. If the nonlinear relationship was deduced using deep learning, a dual energy image could be generated from single energy chest radiography without double exposures.

They had utilized chest radiograms in training (lung image database consortium (LIDC-IDRI)) database [22]. Their training data utilized in this study were a single energy and dual energy chest radiogram pair. They utilized single energy chest radiogram and dual energy soft tissue free image. Deep learning model is a U-net based model and they added a shortcut connection between convolution layers. To optimize such a learning model, they had utilized the adaptive momentary estimation (ADAM) optimization method.

The virtual dual energy [22] bone free chest radiogram was obtained by subtracting the predicted dual energy soft tissue free chest radiograms from the conventional single energy chest radiogram. Kenji Suzuki developed pixel based device mechanism using medical image processing which avoids error caused by inaccurate feature calculation and segmentation while classifying objects into certain classes [23]. Takeshi Kobayashi, Xin-Wei Xu, Heber MacMahon, Charles E. Metz, Kunio Doi evaluate the consequence on nodule output by utilizing ROC analysis with two diverse techniques involved in computer aided diagnosis scheme [24]. Donghoon Lee, Hwiyoung Kim, Byungwook Choi, Hee-Joung Kim developed a deep learning which reduces double exposure with improvement of diagnostic accuracy [25].

In this work, MANN based soft tissue technique has been expanded with JSRT image set in support of subtle nodule recognition. It will facilitate a proposed computer aided detection scheme without double x-ray exposures.

## Methods

### Database of X-ray image

A 247 image set has been downloaded from the Japanese Society of Radiological Technology (JSRT) (http://db.jsrt.or.jp/eng.php). From that, 140 abnormal and 93 normal images were selected. Detail is made available in Table 2.

**Table 2:** Allotment of nodules in the JRST database based on nodule size

	Size(mm) categories			Total
	Small	Medium	Large	
Tremendouslysubtle	2	18	5	25 (16.2%)
Very subtle	3	16	10	29 (18.8%)
subtle	4	29	17	50 (32.5%)
Relatively observable	1	20	17	38 (24.7%)
observable	0	5	7	12 (7.8%)
**Pathology**				
Benign	7	34	13	54
Malignant	3	54	43	100

Selected images have been subjected to nodule detection with absence in opaque portions. These sizes were 2048 × 2048 pixels. All nodules in this database were validated by computed tomography and their location was verified by chest radiologists. A digitized image having 12 bits with a pixel quality of 2048 × 2048. A pixel size was 0.175 × 0.175 mm. Subtle nodule may be divided into five stages which are tremendously subtle, very subtle, subtle, observable, relatively observable.

A MANN based soft tissue technique has been created for discerning precise opacity from other opacities in chest radiography. So, it is utilized to differentiate subtle nodules. This technique was required when it has acquired equipping image by rib suppression and was evaluated by 233 images. The allotment of nodules in the JRST database was based on its size and précised in Table 2.

Shiraishi et al. [26] have eliminated cases in this study comprising lung nodule in opaque scenarios for x-ray image that match up to the retro-cardiac as well as sub-diaphragmatic areas of the lung. However, 7.6% (76/1000) of these scenarios belong to these areas. Opaque scenarios represent 9.1% (14/154) of the JRST dataset.

### Existing computer aided detection scheme

At a University of Chicago Hospital in a Department of Radiology (https://radiology.uchicago.edu/about/early-years), x-rays were acquired utilizing a single exposure based dual energy radiography system. Original image dimensions was 1760 × 1760. This dimension is reduced to 512 × 512 by utilizing sub sampling for a considerable decrease in computation time.

#### Overview

First module employs an imaging pipeline [27] which contains lung separation from other arrangements by utilizing x-ray with an area being suspected as an abnormality. With this module, the system extracts 65 × 65 square areas for considering suspicious point positioned within middle area. Because it employs pixel-based method, every pixel situated in square area were believed as system inputs. The intensity values fall within these inputs were extorted and stored in a database was utilized to train system at second module. The database was alienated into a number of subcategories, and the information offered in these subcategories would be utilized for the training as well as for testing the results. In a second module, neural network was equipped through input categories which are named as statistical feature based inputs and pixel based inputs.

**Fig. 1:**
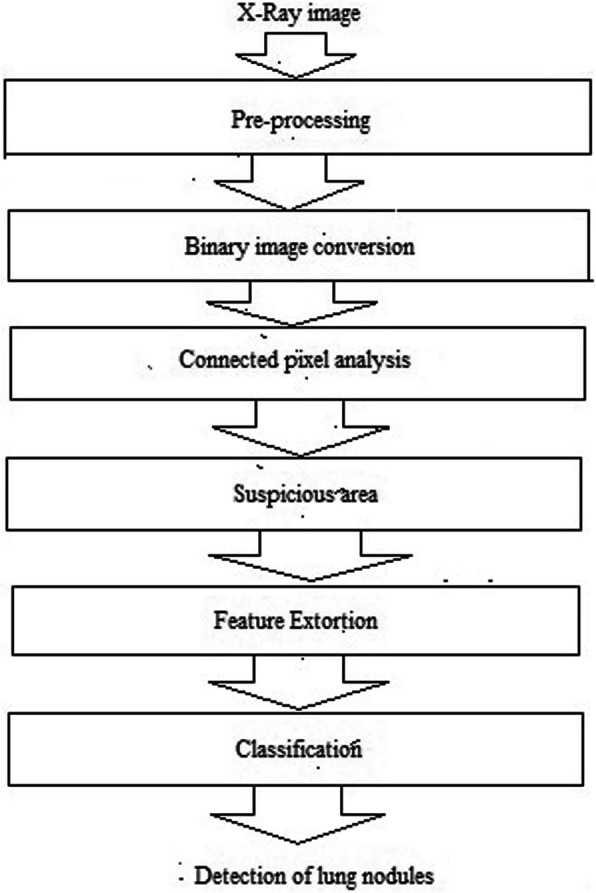
Existing computer aided detection scheme

An existing computer aided detection scheme include four major steps which is represented in Fig. 1: A) pre-processing B) Binary image conversion and connected component analysis C) feature extortion D) classification.

**Fig. 2:**
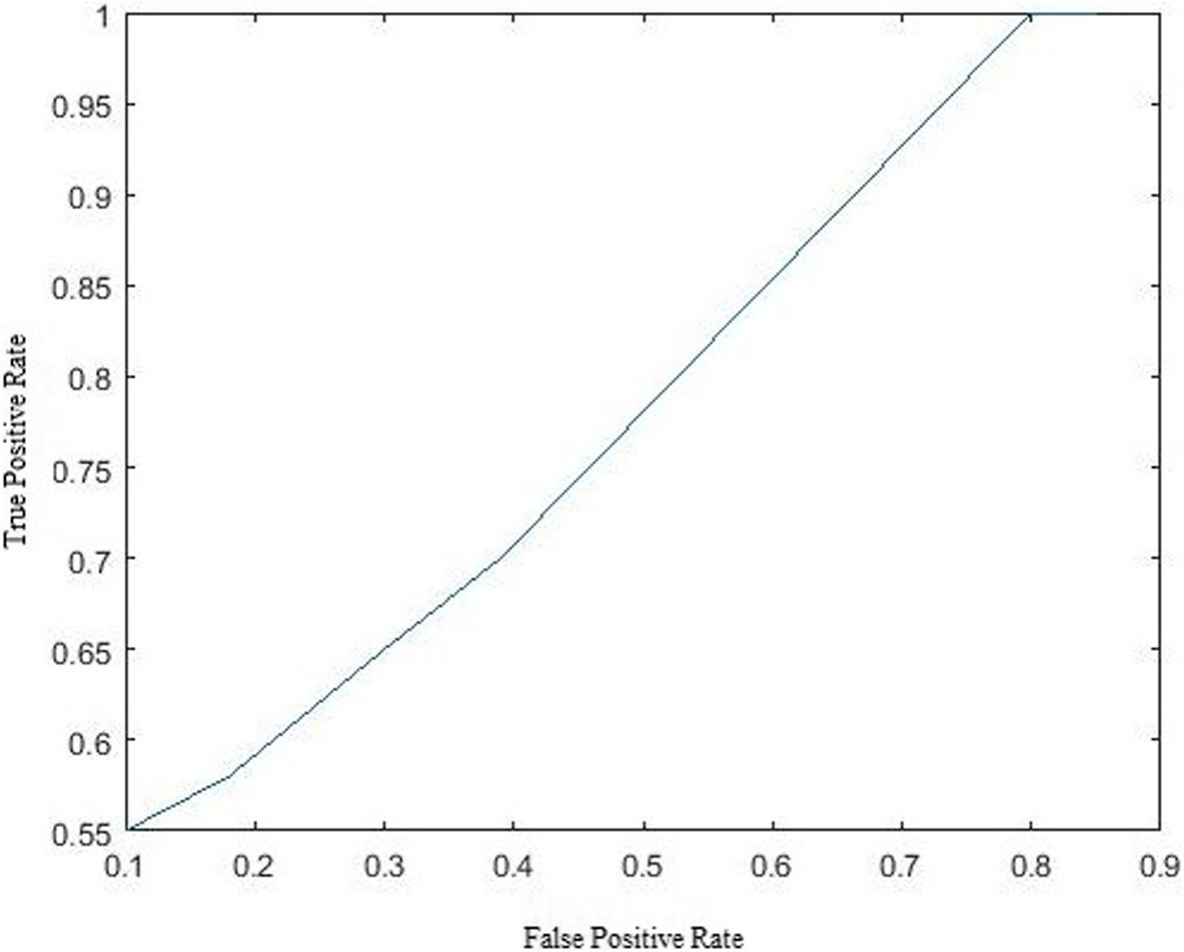
ROC Curve

It contains two modules which were depicted inside Fig. 2.

##### Pre-processing

When they utilized median filtering technique during preprocessing step, the poor contrast effect had eliminated. A low frequency image was created by substituting pixel value with median pixel value over a square area as 5 × 5 pixel centered at pixel location. Sharpening and histogram equalization techniques were utilized in the direction of enhancing image contrast.

##### Binary image conversion and connected component analysis

Binary image conversion [27] has been done to make a computation apt for threshold procedure. By utilizing threshold image, lung masks were prepared through active shape models. However, these masks may be utilized in the connected component analysis during scope identification while user selecting suspicious points [27]. So, lung mask was utilized to group pixel region as an element. i.e., every pixel region having a related element was related to each other.

The criterion for x-ray image enclosure using JSRT image set were: (1) nodule absence bigger than 35 mm, (2) suspicious nodule absence that were not launched by CT examination, and (3) nodule absence with margin that might not be established by a radiologist. The subtlety holded within this image set are clustered into five categories, namely, observable, relatively observable, subtle, very subtle and tremendously subtle. These categories have been described by expert radiologists which takes into account size, contrast, and anatomical position of the lesion.

##### Feature extortion

Fourteen features were extorted from the above method (Binary image conversion and connected component analysis) and listed in Table 3.

**Table 3:** Features extorted using existing computer aided detection scheme

S.No.	Extorted feature
1	Contrast value
2	Correlation value
3	Energy value
4	Homogeneity value
5	Gray level
6	Mean
7	Standard Deviation
8	Entropy
9	Circularity
10	Uniformity
11	Smoothness of the intensity
12	Skewness of the histogram
13	Area
14	Perimeter

The circular index of each connected module M_i_ is defined as

1 $$ {\mathrm{M}}_{\mathrm{i}}=4{\mathrm{JIA}}_{\mathrm{i}}/{{\mathrm{R}}_{\mathrm{i}}}^{\wedge }2 $$

Where *A*_i_ is the area (nodule) of each image in JSRT image set, R_i_ is the perimeter (nodule) of each image in JSRT image set. It is calculated based on the area and the perimeter. If a connected module exhibits a circular index nearer to 1, then there is a high probability of nodule consideration. After recognizing a region that illustrates a high probability of being a nodule, this scheme proceeds to the second phase of their algorithm to train the classification.

##### Classification

A neural network having one hidden layer of 1000 neurons and an input layer of 10 neurons to hold the first and second order textures were utilized at the training phase. Based on the utilization of the training phase, subtle nodules were grouped into five categories which are named as observable, relatively observable, subtle, very subtle and tremendously subtle. It is based on size, contrast, and anatomical position of the nodule.

### Creation of MANN based soft tissue technique

**Fig. 3:**
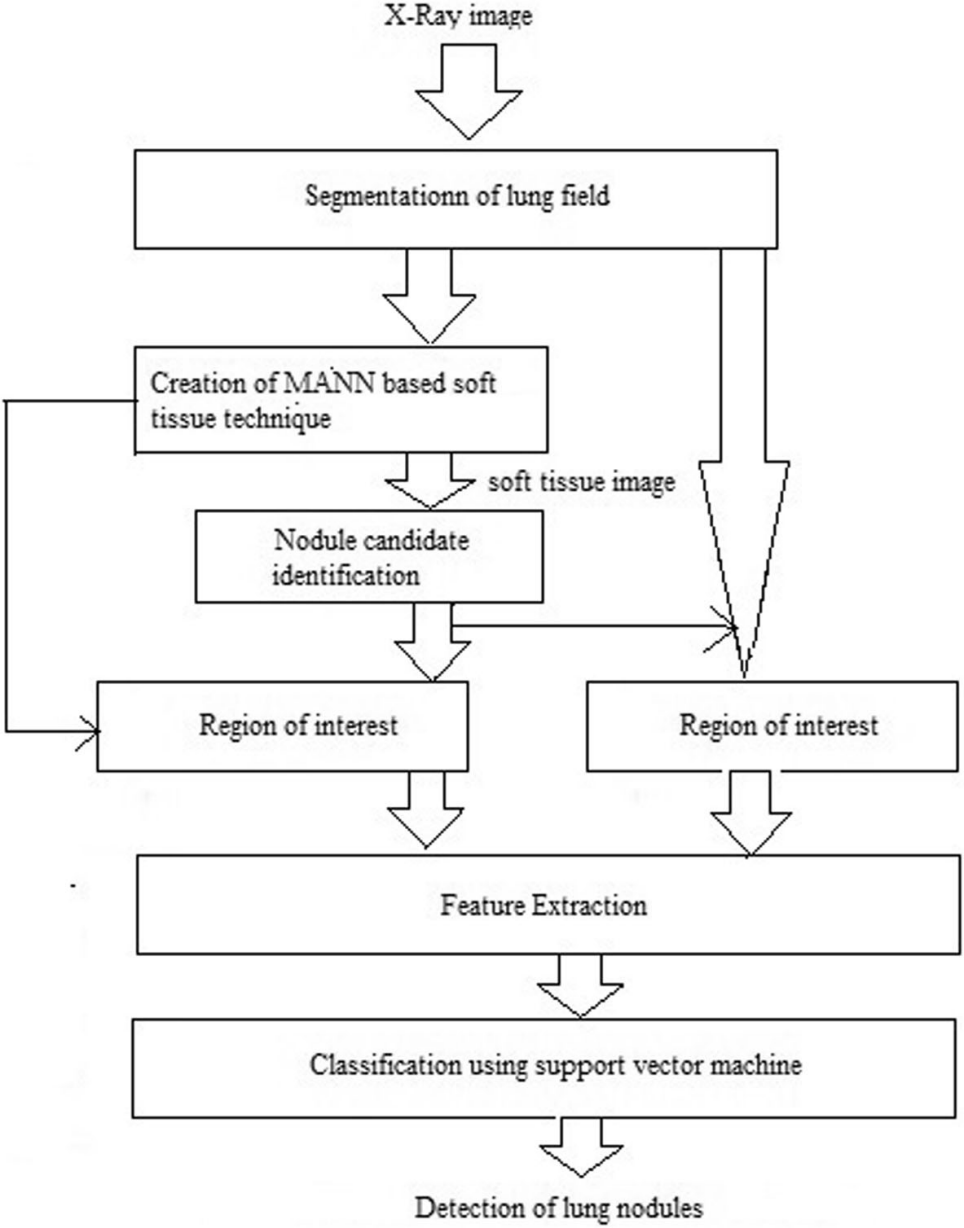
Proposed computer aided detection scheme

In the radiography field, MANN filter [28, 29] was involved. However, this was vital to discriminate precise opacity from other opacities. MANN based soft tissue technique was created (described in eq. 9) by utilizing x ray. It was used as rib and clavicle suppressed [30] form. Figure 3 show soft tissue technique creation using x-ray. MANN [31], nonlinear filter has equipped. Bone imaging has been acquired by means of dual energy radiography methods and it was utilized as equipping image. Equation (2) represents a mapping of input vector utilizing neural network.

The MANN contain linear-output ANN regression model which is able to handle image data directly.

2 $$ \mathrm{f}\left(\mathrm{u},\mathrm{v}\right)=\mathrm{NN}\ \left({\mathrm{a}}_{\mathrm{u},\mathrm{v}}\right) $$

Where a_u,v_ = {g(u-i,v-j) | u-i, v-j є R_s_} is an input vector to MANN which represent sub region, f(u,v) represent an estimate of a teaching value. Equation (3) represents an actual function of rib suppression between input vectors and training values. R_s_ and R_t_ denote sub and training region.

3 $$ \left\{\mathrm{a}\left(\mathrm{u},\mathrm{v}\right),\mathrm{T}\left(\mathrm{u},\mathrm{v}\right)|\ \mathrm{u},\mathrm{v}\in {\mathrm{R}}_{\mathrm{T}}\right\}=\left\{\ \left({\mathrm{a}}_1,{\mathrm{T}}_1\right)\ \left({\mathrm{a}}_2,{\mathrm{T}}_2\right)\dots \dots \left({\mathrm{a}}_{\mathrm{N}},{\mathrm{T}}_{\mathrm{N}}\right)\ \right\} $$

Where T (u, v) is a training image and N is pixel number in training region._._ For a sole MANN, rib holding different frequencies maintains complex suppression due to limited ability. With an intention of conquering this issue, multi resolution decomposition/composition techniques were applied. First lower resolution image G_L_ (u, v) acquired from higher resolution image G_H_ (2u, 2v) by executing down sampling with average, i.e., four pixels replaced by a mean value of four pixels represented by an eq. (4)

4 $$ {\mathrm{G}}_{\mathrm{L}}\left(\mathrm{u},\mathrm{v}\right)=\frac{\left(1/4\right)\sum {\mathrm{G}}_{\mathrm{H}}\left(2\mathrm{u}-\mathrm{i},2\mathrm{v}-\mathrm{j}\right)}{\mathrm{u},\mathrm{v}\in {\mathrm{R}}_{22}} $$

Where R_22_ represent 2 × 2 region. The lower resolution area replaced four regions having the same value through up sampling, were represented in an eq. (5) as follows:

5 $$ {G}_L^U\left(u,v\right)={G}_L\left(u/2,v/2\right) $$

6 $$ \mathrm{S}={\boldsymbol{G}}_{\boldsymbol{L}}^{\boldsymbol{U}}\left(\boldsymbol{u},\boldsymbol{v}\right) $$

Then, enlarged lower resolution region is subtracted from higher resolution region shown in eq. (6) and (7)

7 $$ {\mathrm{D}}_{\mathrm{H}}\left(\mathrm{u},\mathrm{v}\right)={\mathrm{G}}_{\mathrm{H}}\left(\mathrm{u},\mathrm{v}\right)-\mathrm{S} $$

This procedure was performed uninterruptedly in the lower resolution area. Thus, multi resolution area was crafted by using a multi resolution decomposition method. A vital asset of this method is as same as high resolution area G_H_ (u,v) is acquired in eq. (8) is as follows:

8 $$ {\mathrm{G}}_{\mathrm{H}}\left(\mathrm{u},\mathrm{v}\right)=\mathrm{S}+{\mathrm{D}}_{\mathrm{H}}\left(\mathrm{u},\mathrm{v}\right) $$

As a result, preference will be given to multi resolution region. After training of this technique, x-ray image produces bone area which was similar as training bone area. The bone area f_b_(u,v) was created from training neural network. Along with it, lung masking area n(u,v) and weighting parameter w_c_ which was subtracted from the sub region g(u,v) to create soft tissue in eq. (9).

9 $$ \mathrm{f}\left(\mathrm{u},\mathrm{v}\right)=\mathrm{g}\left(\mathrm{u}-\mathrm{i},\mathrm{v}-\mathrm{j}\right)-{\mathrm{w}}_{\mathrm{c}}\mathrm{x}\ {\mathrm{f}}_{\mathrm{b}}\left(\mathrm{u},\mathrm{v}\right)\ \mathrm{x}\ \mathrm{n}\left(\mathrm{u},\mathrm{v}\right) $$

Where f(u,v) denotes the soft tissue having different types of rib contrast using weighting parameter w_c._

To diminish rib-induced false positive and discern nodule overlapping ribs and clavicles, we have included MANN based on soft tissue technique within a proposed computer aided detection scheme.

Major issues faced during existing computer aided detection scheme was toward discerning nodule superimposed with ribs, rib crossings, and clavicles. During rib as well as clavicle suppression in x-ray image, some nodule candidate has missed by soft tissue technique. We identified those nodule candidates which was done for non subtle nodule within x-ray image through the following steps A) lung field extortion utilizing multi-division active shape model (M-ASM) [32] B) region of interest based on an abnormal identification by utilizing clustering watershed technique C) feature extortion D) classification. During this scenario, sensitivity of proposed computer aided detection scheme is lower than the sensitivity of existing computer aided detection scheme for non subtle nodules.

However, sensitivity of existing computer aided detection scheme has progressed by minimum improvement.

### Proposed computer aided detection scheme

**Fig. 4:**
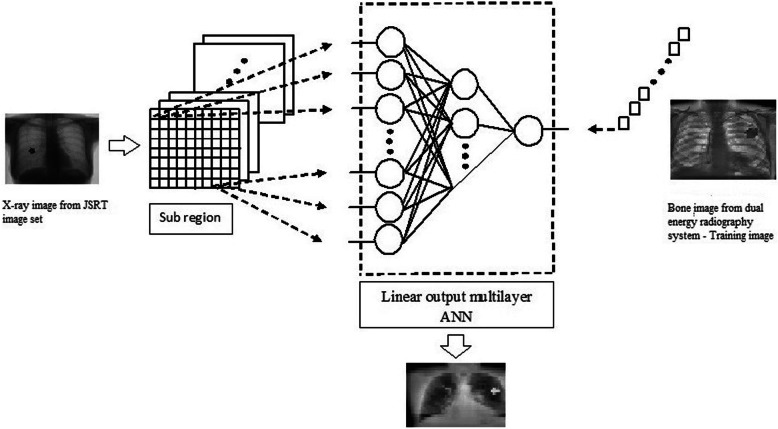
Creation of MANN based soft tissue technique

Figure 4 demonstrates proposed computer aided detection scheme. It incorporates 4 steps: A) lung field extortion utilizing multi-division active shape model (M-ASM) B) region extortion based on an abnormal identification by utilizing clustering watershed method C) feature extortion D) an abnormal categorization utilizing support vector machine.

#### Lung field extortion utilizing multi-division active shape model (M-ASM)

Multi-division active shape model (M-ASM) were permitted during lung field extortion [33] for structural boundary. It determines multiple segments [34] called heart, aorta as well as rib-cage. The node specified active shape model was discovered through each segment for a particular boundary which resulted in a marked improvement in boundary accuracy. After lung field extortion, background trend correction technique based on second order bivariate polynomial function was employed using eq. (10)

10 $$ \mathrm{F}\ \left(\mathrm{x},\mathrm{y}\right)={\mathrm{ax}}^2+{\mathrm{by}}^2+\mathrm{cxy}+\mathrm{dx}+\mathrm{ey}+\mathrm{f} $$

where a, b, c, d, e,f are co-efficients and F (x, y) denotes an image. In this, x and y indicate pixel co-ordinates.

Segmented lung field image applies different gray level morphological open operation [35, 36] forming nodule enhanced images and a nodule enhanced image have modified likelihood map. MANN based soft tissue technique [28] have created after lung field segmentation to suppress rib and clavicle in x-ray image. In Fig. 3, a soft tissue image recognized nodule candidate by utilizing two step nodule enhancement technique (which was done for subtle nodules). Region of interest have identified using soft tissue and x-ray image and feature based on these images are effective. Some nodule had related bone feature, i.e., shape, size, contrast, orientation. However, these features were suppressed using this technique. Due to suppressed feature, identified nodule may be misinterpreted as non nodule in the soft tissue image. To identify such misinterpretation, same feature set may be extorted at the equivalent location in x-ray image [37, 38].

#### Region extortion based on an abnormal identification by utilizing clustering watershed method

Region of interest was identified based on an abnormality by utilizing clustering watershed technique after lung field segmentation. By utilizing a clustering watershed technique [39], the jagged abnormal area was segmented using multiple catchment basin [40, 41]. Every least point was enclosed by it; thus, there were one or more peaks, each of which was included by a cluster of associated pixels that comprised a catchment basin. From the multiple catchment basin, a single abnormal area was concluded by following clustering method: first, primary cluster was included abnormal location (as a point) decided by initial identification step. Next, clusters connected to primary cluster were inserted. Attached clusters were recognized through utilizing least value between peaks in primary cluster.

#### Feature extortion

Sixty two morphological and gray-level-based features were extorted from the region of interest through x-ray and soft tissue image. The features extorted from x-ray and soft tissue image using proposed computer aided detection scheme were listed in Table 4.

**Table 4:** Feature Extortion

S. No	Feature Extortion	Description
1	can.u	Co-ordinates of a nodule candidate(horizontal)
2	can.v	Co-ordinates of a nodule candidate(vertical)
3	can.Grad_1_	Co-ordinates of a nodule likelihood values
4	can.CV_1_	Computed by using gray level values
5	can.Grad_2_	Co-ordinates of a nodule likelihood values
6	can.CV_2_	Computed by using gray level values
7	Shape_1_	Area for a segmented nodule candidate(A_region_)
8	Shape_2_	Short and long axes of an ellipse which are robust to nodule candidate
9	Shape_3_	A_region_/A_convex hull_
10	Shape_4_	[d_candidate- center_ /squarero ot(Shape_1_/Л)]
11	Gray_1_	μ^region^ – μ^surround^ The gray-level feature was estimated using fragmented candidates and their surrounding regions in both pre-processed image and nodule-enhanced image. A surrounding region was constructed by subtracting a candidate region from a dilated candidate region. μ^region^➔ Mean of a region μ^surround^➔Mean of a surrounding region
12	Gray_2_	σ^region^- σ^surround^ σ^region^➔Standard deviation of a region σ^surround^➔Standard deviation of a surrounding region
13	Gray_3_	min^region^-min^surround^ min^region^➔ Minimum value of a region min^surround^➔Minimum value of a surrounding region
14	Gray_4_	max^region^-max^surround^ max^region^ ➔ Maximum value of a region max^surround^➔Maximum value of a surrounding region
15	Gray_5_	Calculated using Gray_1_
16	Gray_6_	Calculated using Gray_2_
17	Gray_7_	Calculated using Gray_3_
18	Gray_8_	Calculated using Gray_4_
19	Grad_1_	$\overline{\mathrm{Gr}}=\left(1/8\right)\sum \limits_{\mathrm{k}=0}^7{\mathrm{Gr}}^{\mathrm{h}}$ $$ {\displaystyle \begin{array}{c}{\mathrm{Gr}}^{\mathrm{h}}=\left[1/{\mathrm{N}}_{\mathrm{h}}\right]\sum \cos \kern0.28em {\alpha}_{\mathrm{mn}}\\ {}\mathrm{mn}\in {\mathrm{region}}_{\mathrm{h}}\\ {}{\mathrm{t}}_1\le {\mathrm{M}}_{\mathrm{mn}}\le {\mathrm{t}}_2\end{array}} $$ where N_h_ is number of pixels in segmented candidate area h and cos α_mn_ denotes likelihood values used in two stage nodule enhancement method
20	Grad_2_	$\upsigma =\surd \sum \limits_{\mathrm{k}=0}^7{\left({\mathrm{Gr}}^{\mathrm{h}}-\mathrm{Gr}\right)}^2$
21	Grad_3_	$=\overline{\mathrm{Gr}}/\sigma$ $\overline{\mathrm{Gr}}$ denotes gradient feature
22	Surface_1_	λ_min_ Segmented candidate area in nodule enhanced image was robust to fourth order bivariate polynomial. The principal curvatures was computed at highest elevation point in the candidate region.
23	Surface_2_	λ_max_
24	Surface_3_	λ_min_ λ_max_
25	Texture_1_	∑ [C(i,j)^2^] ij C(i,j) ➔Co-occurrence matrix computed over neighboring pixel and a summation range from minimum to maximum pixel value in pre-processed image
26	Texture_2_	∑ (i-j)^2^C(i,j)^2^ ij C(i,j) ➔Co-occurrence matrix calculated over neighboring pixels and a summation range from the minimum to the maximum pixel value in the pre-processed image.
27	Texture_3_	Calculated based on Texture_1_ and Texture_2_
28	Texture_4_	Calculated based on Texture_1_ and Texture_2_
29	Texture_5_	Calculated based on Texture_1_ and Texture_2_
30	Texture_6_	Calculated based on Texture_1_ and Texture_2_
31	False Positive	Loverlap / Lregion Where Lregion is length of boundary of a candidate area and Loverlap is number of pixels on boundary that overlap edge chain.

#### Classification

After nodule [42] area extortion, feature extortion [43] have given as an attempt to non linear support vector machine [44] (SVM) for categorizing abnormality. Based on feature extortion, a common size of an abnormal area (17.8 mm) was detected. An SVM classifier [44] has trained/tested by applying cross-validation experiment and FROC analysis has attained [45, 46].

## Results

Here, proposed computer aided detection scheme has been demonstrated. First, MANN based soft tissue technique was created. Next, the soft tissue image having different rib contrast was plotted using sensitivity in favor of finding peak value.

### MANN based soft tissue technique training

Four images from JSRT database have used to train MANN. one was normal while other three were abnormal. ROC curve is shown in Figure 1. Massive artificial neural network size has 9 × 9 pixels.

**Fig. 5:**
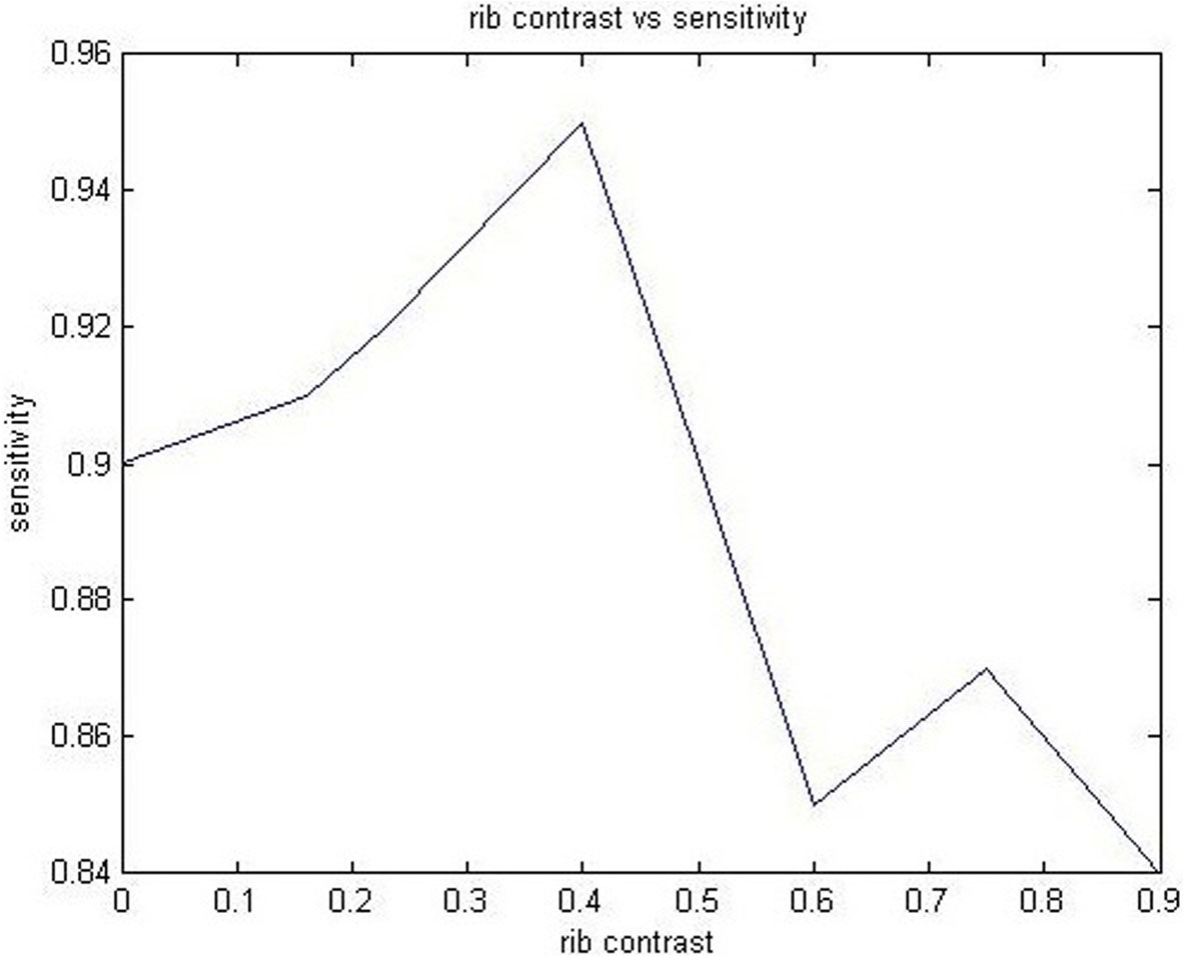
Rib contrasts vs sensitivity

It was enough to wrap rib width in the image. The limited figure of bone images was utilized in three layered, massive training artificial neural network to restrain rib where input, hidden and output units was 81, 20 and 1 respectively. Figure 5 demonstrates plotted rib contrast values using soft tissue images. It is explained in the next section.

### Nodule candidate identification

**Fig. 6:**
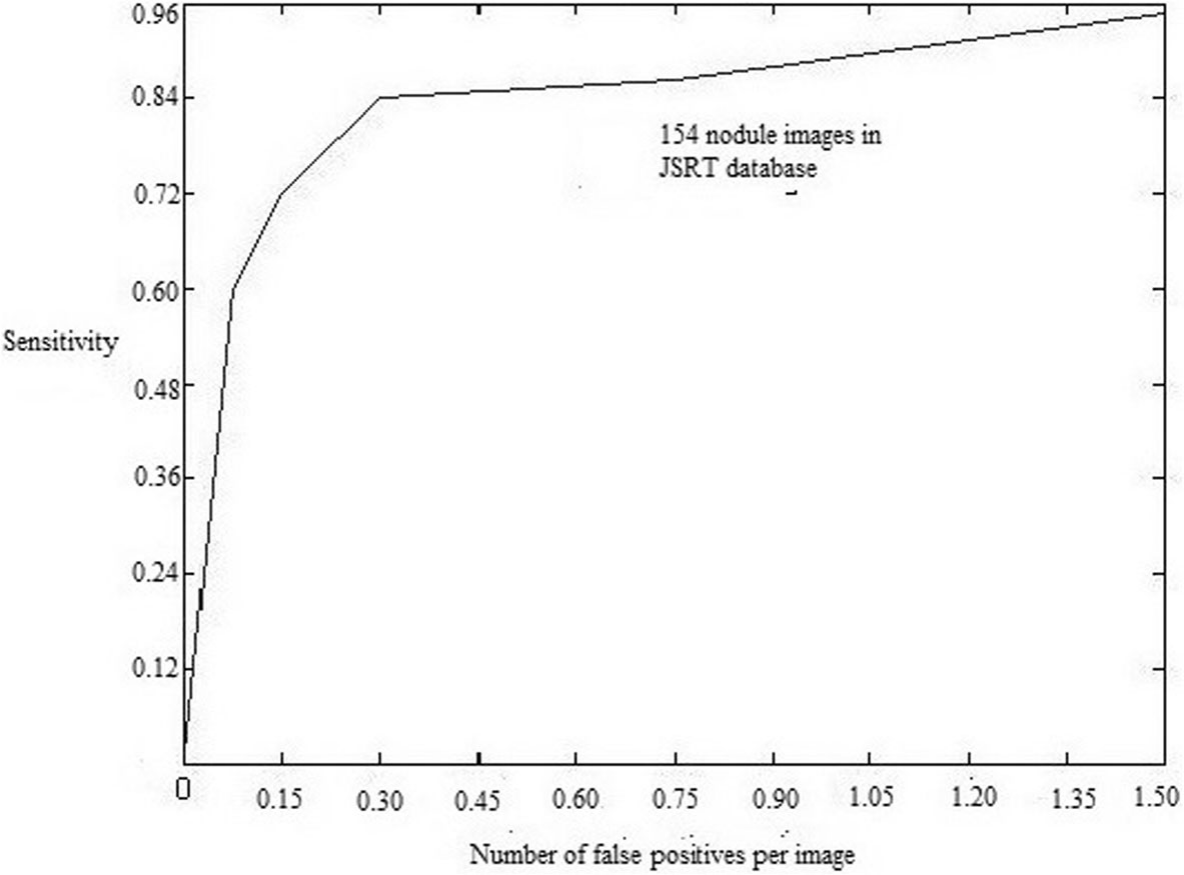
Sensitivity vs number of false positive per image

We subtract bone image from the novel x-ray image as per eq. (10) in Section 2.3. From this, we obtain a soft tissue image. There was a factor Mc to regulate rib disparity in soft tissue images. A rib disparity [47] is a factor obtain from a different soft tissue image by the use of the weighting parameter(eq. (10)) and it’s range lies between 0 and 1. In Fig. 5, highest sensitivity value (95%) is concluded while rib (contrast) parameter M_c_ was 0.4. As in soft tissue image, most nodules were identified in different rib contrast during a candidate identification step. In a plotted graph, 84% candidates have max code (nodule likelihood) values represent probability map were utilized toward an origin point. The nodule candidate identification in MANN based soft tissue technique utilizing JSRT image set was achieved 95% (135/140) sensitivity. In Fig. 6, Number of false positives per image vs Sensitivity were plotted. False positive of 1 were obtained by using 84% sensitivity for 154 nodule images. The values of features extracted from x-ray and soft tissue image using proposed computer aided detection scheme are shown in Table 5.

**Table 5:** Features values from x-ray and soft tissue image using proposed computer aided detection scheme

Feature Extortion	Values in x-ray image	Values in soft tissue image
can.u	6	7
can.v	7.5	8.5
can.Grad_1_	4.5	5.5
can.CV_1_	0.65	0.75
can.Grad_2_	6.7	7.7
can.CV_2_	7.2	8.2
Shape_1_	7	8
Shape_2_	6.6	7.6
Shape_3_	4.5	5.5
Shape_4_	5.8	6.8
Gray_1_	7	8
Gray_2_	8.2	9.2
Gray_3_	8.4	9.4
Gray_4_	9.2	8.2
Gray_5_	7	8
Gray_6_	8.2	9.2
Gray_7_	8.4	9.4
Gray_8_	9.2	9.2
Grad_1_	37	47
Grad_2_	30	40
Grad_3_	1.23	1.175
Surface_1_	25	35
Surface_2_	27	37
Surface_3_	675	1295
Texture_1_	30	40
Texture_2_	32	42
Texture_3_	34	44
Texture_4_	36	46
Texture_5_	38	48
Texture_6_	40	50
False Positive	2.5	1

Table 6 indicates a sensitivity and false positive of several computer aided detection schemes which was utilized JSRT image set. Wei et al. [48] gave information about their CAD scheme which attained 80% sensitivity and 5.4 FPs per image by means of utilizing JSRT database. Due to large amounts of false positive (5.4), radiologist accuracy in identifying lung nodule was not progressed. Hardie et al. gave information about their CAD schemes which was marked 63% nodules in JSRT image set and 2 FPs per image [50]. Their concert was considered by utilizing 25 mm distance decision for finding.

**Table 6:** Performance comparison of several existing computer aided detection systems which used JSRT Database

Author	Sensitivity	FPs/image	Methodology	Classifier	Database
Wei et al. [48]	80% (123/154)	5.4 (1333/247) (less accuracy due to high FPs)	Forward stepwise selection	Fisher linear discriminant	All abnormal as well as normal image inside JSRT(247)
Coppini et al. [49]	60% (93/154)	4.3 (662/154)	Neural network filter	Fisher linear discriminant	All nodule image in JSRT(154)
Schilham et al. [13]	51% (79/154) 67% (103/154)	2 (308/154) 4 (616/154)	Image filtering Based on Regression	Fisher linear discriminant	All nodule image in JSRT(154)
Hardie et al. [50]	80% (112/140) 63% (88/140)	5.0 (700/140) 2 (280/140)	Active shape model and new weighted multi- scale conver gence-index	Fisher linear discriminant	Nodule image in JSRT(140)
Chen et al. [31]	71% (100/140)	2 (466/233)	Computer aided detection using neural filter	Support Vector Machine (SVM)	Nodule and Normal image in JSRT(233)
Proposed MANN based soft tissue technique	72.85% (102/140)	1 (233/233)	MANN for rib suppression	Support Vector Machine (SVM)	Nodule and normal image in JSRT(233)

out true positive (TP) recognitions*.* Concert of our CAD system with MANN based soft tissue technique (72.85%) has substantially higher than Chen reported scheme [31].

## Discussion

By utilizing dual-energy subtraction [51, 52] technique, it was very complex task in the direction of gathering larger radiation dose. MANN based soft tissue technique has the possibility toward improving sensitivity with specificity which was buried as a consequence of suppressing rib with a discriminating nodule contained by an another regular anatomic structure. For an obscuring bone, single exposure based dual energy subtraction technique [44] had addressed. By utilizing this technique, the soft-tissue image was created. When we use this technique, sensitivity and specificity were improved. A fixed number of hospitals utilize radiography systems (www.ijetae.com, www.ajronline.org) [53, 54] by dual-energy subtraction, because dedicated tools for acquiring dual-energy X-ray exposures [55] was required.

As within x-ray image, we observe nodule candidate during rib contrast [56] parameter variation. a false positive was noticed. In our approach, MANN based soft tissue technique was utilized in direction for building soft tissue image. It was trained using four x-ray images (one was a normal image while other three was nodule image) from JSRT image set and corresponding bone images. Highest rib contrast factor has been observed. We utilize this trained MANN on behalf of restraining ribs and clavicles.

In a proposed CAD, MANN based soft tissue technique has integrated 2 diverse images (bone, x-ray image) together and extorted feature set using soft tissue image after lung field segmentation. During this technique, a constant rib contrast factor was preferred which was represented as maximum peak in feature recognition in favor of every abnormal cases utilizing soft image for reducing false positives which are caused by ribs.

## Conclusion

Here, the proposed computer aided detection (CAD) scheme using MANN based soft tissue technique is being widened as 72.85% sensitivity and 72.96% accuracy after sub region identification. Hence, the subtle nodule was detected using a nonlinear filter. In this work, the nonlinear filter was MANN. It is a promising method for radiologists to recognize an abnormality by x-ray images from JSRT image set. By utilizing MANN filter, false positive of the proposed CAD scheme have diminished to 1 which was lower than previous works.

## Data Availability

The dataset that support the findings and conclusion of this study are publicly available.

## Abbreviations

CAD: Computer aided diagnosis
MANN: Massive Artificial Neural Network
JSRT: Japanese society of radiological technology
FP: False Positive
